# mHealth Engagement for Antiretroviral Medication Adherence Among People With HIV and Substance Use Disorders: Observational Study

**DOI:** 10.2196/57774

**Published:** 2024-12-20

**Authors:** Ranran Z Mi, Ellie Fan Yang, Alexander Tahk, Adati Tarfa, Lynne M Cotter, Linqi Lu, Sijia Yang, David H Gustafson Sr, Ryan Westergaard, Dhavan Shah

**Affiliations:** 1 Department of Communication, Media and Journalism Kean University Union, NJ United States; 2 School of Communication Illinois State University Normal, IL United States; 3 Department of Political Science University of Wisconsin-Madison Madison, WI United States; 4 Yale School of Medicine New Haven, CT United States; 5 School of Journalism and Mass Communication University of Wisconsin-Madison Madison, WI United States; 6 Industrial Engineering and Preventive Medicine University of Wisconsin-Madison Madison, WI United States; 7 Department of Medicine School of Medicine and Public Health University of Wisconsin-Madison Madison, WI United States

**Keywords:** information and communication technologies, ICTs, mHealth, medication adherence, HIV care, antiretroviral therapy, substance use, social support, patient management, health disparities, information technology, communication technology, mobile health, app, clinic, United States, participants, mobile phone

## Abstract

**Background:**

Despite the increasing popularity of mobile health (mHealth) technologies, little is known about which types of mHealth system engagement might affect the maintenance of antiretroviral therapy among people with HIV and substance use disorders.

**Objective:**

This study aimed to use longitudinal and detailed system logs and weekly survey data to test a mediation model, where mHealth engagement indicators were treated as predictors, substance use and confidence in HIV management were treated as joint mediators, and antiretroviral therapy adherence was treated as the outcome. We further distinguished the initiation and intensity of system engagement by mode (expression vs reception) and by communication levels (intraindividual vs dyadic vs network).

**Methods:**

Tailored for people with HIV living with substance use disorders, the mHealth app was distributed among 208 participants aged >18 years from 2 US health clinics. Supervised by medical professionals, participants received weekly surveys through the app to report their health status and medication adherence data. System use was passively collected through the app, operationalized as transformed click-level data, aggregated weekly, and connected to survey responses with a 7-day lagged window. Using the weekly check-in record provided by participants as the unit of analysis (N=681), linear regression and structure equation models with cluster-robust SEs were used for analyses, controlling within-person autocorrelation and group-level error correlations. Racial groups were examined as moderators in the structure equation models.

**Results:**

We found that (1) intensity, not initiation, of system use; (2) dyadic message expression and reception; and (3) network expression positively predicted medication adherence through joint mediators (substance use and confidence in HIV management). However, intraindividual reception (ie, rereading saved entries for personal motivation) negatively predicts medication adherence through joint mediators. We also found Black participants have distinct usage patterns, suggesting the need to tailor mHealth interventions for this subgroup.

**Conclusions:**

These findings highlight the importance of considering the intensity of system engagement, rather than initiation alone, when designing mHealth interventions for people with HIV and tailoring these systems to Black communities.

## Introduction

### Background

Mobile health (mHealth) technology has emerged as a promising avenue in health care for addressing the complex challenges faced by people with HIV [1,2]. This population often grapples with their stigmatized condition while struggling with substance use disorders (SUDs) as an additional burden [3]. Substance use may hamper adherence to antiretroviral therapy (ART), which suppresses HIV viral load, thus reducing HIV transmission and preventing the health consequences of HIV infection [4]. As current human rights frameworks advocate that global HIV prevention needs to consider supporting individual treatment [5,6], this study focuses on understanding the potential benefits of mHealth technologies for people with HIV living with SUDs for maintaining ART medication adherence.

Most mHealth apps leverage the technological affordances of mobile phones to improve the health of users. The “anytime-anyplace” mobile access enables wide reach and improves how we coordinate and communicate [7-10]. In addition, mobile log data help identify evidence-based strategies for health intervention and message delivery [11-15]. Researchers have conducted studies that differentiate message expression from message reception across various communication levels and found that composing one-on-one messages is associated with greater perceived bonding, and that drafting public forum posts is linked to decreased risky drinking behaviors [16].

This study aims to understand how mHealth systems can influence adherence to ART, defined as consistent and proper compliance with the prescribed medication regimens for HIV viral load suppression. Numerous mobile technologies have been developed for interventions to enhance medication adherence among people with HIV [17]. For example, some focused on the effectiveness of electronic adherence monitoring (EAM) devices that remind patients to take pills [18]. Others adopted text message services to deliver motivational and skill-building content [19], launched social media campaigns that promote adherence and testing [20], and designed mobile games to make medication adherence enjoyable and goal-oriented [21]. However, there is a lack of understanding of how these interventions are beneficial for medication adherence. For instance, it is unclear how different communicative behaviors supported by the system would shape medication adherence and how within-person and group-level attributes should be considered when analyzing the system logs data.

To bridge this gap, we investigated both the initiation and intensity of mHealth engagement at the network (one-to-many), dyadic (one-to-one), and intraindividual (self-to-self) communication levels to predict ART medication adherence among people with HIV. In addition, we tested 2 sets of indirect pathways—different forms of substance use as risk factors and confidence in HIV management as a protective factor—that potentially mediated the relationships between mHealth engagement and ART medication adherence on a weekly basis. To examine these relationships, we used linear regression and structural equation models (SEMs) with cluster-robust SEs (CRSEs) to control for within-person autocorrelation and group-level error correlations. Finally, this paper explores whether the mediation model differs between Black and White populations and provides insights into designing and implementing mHealth interventions that promote medication adherence for different racial communities.

### Medication Adherence and mHealth

Medication adherence has become a key factor in both individual health and public health efforts to control the spread of the virus. Traditional interventions have often fallen short in engaging and retaining people with HIV in care due to various barriers, such as stigma, limited access to health care facilities, and social isolation [22,23]. Interventions based on mHealth, including smartphone apps and text messaging systems, have shown promise in bridging these gaps. Studies have also shown that mobile apps are feasible and acceptable for use among people with HIV [24].

Although generally seen as providing social support, education, and monitoring, mHealth platforms can provide a wide range of distinct communication options: journal writing, one-to-one messaging, and group discussion posting [25,26]. Scholars have also theorized that distinct health outcomes are associated with message reception (ie, viewing existing content) and expression of messages (ie, sharing one’s own thoughts and feelings) at the intraindividual, dyadic, and network communication levels [16].

### Message Reception and Expression at Each Communication Level

Message reception and expression likely function differently in the process of health behavior change and, more importantly, may trigger specific relational and psychological processes that occur when communicating, depending on the intended audience [16].

At the intraindividual level, expression occurs whenever individuals actively generate new, personal content (eg, creating a journal entry), whereas reception refers to reflecting on self-generated content (eg, reading one’s own previous writing). Pennebaker’s [27] “expressive writing” research highlights the potential benefits of intraindividual expression, specifically through the “release effect” and cognitive processing. On the other hand, self-regulation [28,29] and self-perception [30] frameworks provided alternative accounts, emphasizing the potential benefits of intraindividual reception, which involves observing one’s behavior and making necessary recalibration.

At the dyadic level, reception and expression occur when individuals receive and send, respectively, one-on-one messages. According to social penetration theory, dyadic expression is important because self-disclosure is the main driver for building and maintaining intimate relationships [31], and self-disclosure is most pronounced in dyads [32]. Dyadic reception also matters, according to optimal matching theory, as the help received and solicited in one-on-one messaging is more responsive and customized to specific individual needs [33].

At the network level, expression means crafting a message that can potentially be seen by many readers, whereas reception means viewing content available within such public forums. Network expression holds importance because, according to the concept of public commitment [34], expressing oneself to a group can compel individuals to adhere to their announced plans in order to maintain their reputation. Immersion in shared experiences and the sway of group norms provide potential explanations for why network reception might play a role [35]. By comparing the effects of system use at these distinct communication levels, as opposed to treating communication as a single entity, we can gain a more accurate understanding of the interactions within digital health interventions.

Patterns of human-app interactions can be understood in terms of the initiation and intensity of system use. Initiation indicates activation of system engagement on a given day, with regular initiation indicating consistency of use, while intensity reveals the depth of engagement on that day and the degree of penetration into system use. Studying human-technology interactions with attention to this distinction provides valuable insight into health intervention programs. For example, the level of engagement (ie, intensity), such as time spent per log-in, was more associated with intervention effects in comparison with the number of modules completed (ie, initiation) [36,37]. Numerically, mobile logs induce a significant volume of zero entries when users do not engage with the system, which must also be considered in the analysis. Distinguishing between these two metrics helps probe into subtle features of mHealth engagement: (1) determining whether an individual used a feature on a given day–which is binary, and (2) measuring the intensity of the engagement once initiated–which is continuous. This operationalization also echoes what digital media researchers refer to as the “session of use,” meaning the extent of engagement users spend on a specific URL once they activate the interface [38]. Over time, initiation can be seen as a habitual repetition of engagement as the user consistently activates the feature. On the other hand, intensity represents the depth of use during the activated session. Although the initiation of system use is a prerequisite for an mHealth intervention, the intensity or depth of engagement, such as “lasting longer than 10 minutes”, maybe just as critical in delivering the intervention [36,37].

We hypothesize that the initiation and intensity of message exchange, including reception and expression, at the 3 communication levels will predict ART medication adherence. As mHealth is designed to improve health, we hypothesize that engaging with the communication features will predict enhanced medication adherence.

- H1: The initiation of message reception at the (1) intraindividual, (2) dyadic, and (3) network level positively predicts ART medication adherence.
- H2: The intensity of message reception at the (1) intraindividual, (2) dyadic, and (3) network level positively predicts ART medication adherence.
- H3: The initiation of message expression at the (1) intraindividual, (2) dyadic, and (3) network level positively predicts ART medication adherence.
- H4: The intensity of message expression at the (1) intraindividual, (2) dyadic, and (3) network level positively predicts ART medication adherence.

### SUDs as Risk Factors

Although mHealth interventions have shown significant potential in improving medication adherence, it is crucial to address potential risk factors, notably SUDs, which are linked to poor medication adherence. The misuse of a range of substances, including opioids, alcohol, and stimulants, poses substantial threats and could undermine the effectiveness of mHealth interventions [39,40].

SUDs can potentially mediate the relationship between mHealth engagement and medication adherence because mHealth engagement translates into a reduction of substance use, which eventually supports medication adherence. Past mHealth studies have examined whether mHealth interventions can potentially lead to a reduction in craving and substance use [41]. Such interventions can mitigate the challenges associated with SUDs through craving management, coping assistance, and tailored feedback and reminders. Reduction in substance use eventually improves medication adherence and health outcomes; a large body of evidence consistently shows that users of hard drug (eg, cocaine, heroin) and those with hazardous alcohol use report higher ART nonadherence as well as a greater chance of AIDS progression or death [4,42,43].

Thus, we hypothesize that the relationship between mHealth engagement and medication adherence will be mediated by patients’ substance use, specifically their use of (1) heroin or opioids, (2) alcohol, and (3) stimulants. We also hypothesize that mHealth engagement predicts less substance use, which will, in turn, be associated with higher medication adherence.

- H5: The relationship between the engagement with mHealth and ART medication adherence will be mediated by substance use, including (1) heroin or opioids, (2) alcohol, and (3) stimulants.

### Confidence in HIV Management as a Protective Factor

Confidence in HIV management serves as a protective factor that may encourage people with HIV to adhere to ART treatment and potentially enhance the effectiveness of mHealth interventions. Confidence in HIV management denotes a patient’s belief in the efficacy of their health care regimen, the trust they vest in their health care providers, and their self-care capability.

Confidence in one’s HIV management can also potentially mediate the relationship between mHealth engagement and medication adherence. Research has shown patients who regularly interact with mHealth apps may develop a sense of self-efficacy and a stronger belief in the efficacy of their treatment. Likewise, confidence in HIV management may significantly motivate ART medication adherence. Research shows that individuals who have confidence in the care they are receiving are more likely to follow their prescribed medication regimens diligently [44,45]. Patients confident in their HIV management are more likely to overcome barriers and prioritize medication adherence, improving health outcomes.

As mHealth interactions enhance confidence in HIV management, patients may become more motivated and empowered to adhere to their medication regimens, ultimately improving their overall health and well-being. Thus, we hypothesize that mHealth engagement will positively predict confidence in HIV management, which will, in turn, be associated with higher medication adherence.

- H6: The relationship between engagement with the mHealth app and ART medication adherence will be mediated by confidence in HIV management.

### Racial Disparities in Health

Racial disparities in health care have long been a concerning and pervasive issue with profound implications for health outcomes across various populations. Racial and ethnic minority groups such as Black and Hispanic populations often experience lower medication adherence rates, with an average of 7.5 percentage points lower than those of the White population [46]. The prevalence of comorbid conditions that intersect with HIV care, such as SUDs, is also highly prevalent in racial and ethnic minority groups [47] while facing unique challenges such as access to addiction treatment [48]. In addition, studies have indicated racial minority individuals had greater medical mistrust as compared with their White counterparts, which potentially hinders treatment discussions with their providers [49].

On the other hand, research has unveiled insights into the differences in mHealth use and preference among various racial groups. According to a recent national survey [50], Black adults exhibit a stronger inclination toward using mHealth for health decision-making, sharing health information, and engaging in discussions with their providers compared with non-Hispanic White adults. This is consistent with past reports indicating the Black US population has historically higher smartphone ownership (83%) than non-Hispanic White populations (74.2%) [51], suggesting the value of mHealth interventions for racial minorities.

Given the potential racial differences in mHealth use, substance use, confidence in HIV management, and health behaviors, we raise 3 research questions (RQs) below that are critical for developing targeted interventions that bridge racial disparities in medication adherence.

- RQ1: For Black participants, which types of mHealth engagement predict ART medication adherence, and through which mediators?
- RQ2: For White participants, which types of mHealth engagement predict ART medication adherence, and through which mediators?
- RQ3: How does the relationship between mHealth engagement and ART medication adherence differ between Black and White participants?

## Methods

### Clinical Setting and Recruitment

Recruitment took place at 2 US health clinics. Patients qualified for the study if they were adults aged >18 years, receiving medical care for HIV infection, and had a history of SUDs. This included those who either (1) actively used alcohol or other drugs within the past year or (2) were currently enrolled in an SUD treatment program. Recruitment methods included (1) posting flyers at two health clinics across three counties where the study took place, (2) project managers contacting social workers and case managers at these clinics to refer patients, and (3) asking physicians to review medical records of upcoming appointments and suggest ART-CHESS to eligible patients. A total of 208 participants were enrolled.

### Data Collection

ART-CHESS was specifically designed for people with HIV living with SUDs and provided vulnerable patients with information resources, communication functions, and health support tools. It was distinguished from other apps by offering interactions in an unstigmatized environment that was also free of advertising and scammers [52].

The installation of the app on their smartphones was mandatory, through which they received weekly surveys capturing health status and medication adherence. Supervised by staff with experience in HIV care delivery settings, the app facilitated communication during critical moments to provide necessary support to participants.

Furthermore, 2 types of data were integrated by the user’s unique ID. The first was system engagement data that were passively collected through the app usage logs. Note that while this paper only included system-use data from communication functionalities, the app provides additional noncommunication features. The second came from the baseline and weekly surveys, which asked about demographic information, substance use, confidence in HIV management, and ART medication adherence.

### Measurement

#### mHealth Engagement

The “clicks,” representing user interaction with app functions, served as a measure of participants’ system engagement. Specifically, the “clicks” can capture every interface change when a user keystroked the mobile screen. It is a type of behavioral measurement to track users’ online navigation patterns and has been widely adopted to study user engagement [53,54]. In this study, the initiation and intensity metrics for 6 communication functions (Table 1) were assessed for the period before weekly surveys. For the initiation metric, “0” was coded when there was no click record for a user per day, while “1” was coded for any day with a click in the servers’ record. As for the intensity metric, the number of clicks was aggregated among users who had engaged with particular system features (ie, initiation=“1”). Notably, the intensity metric cannot be created when the initiation metric is coded as “0.” After the 2 metrics were generated, they were transformed into their squared root format and lagged by 7 days before being incorporated into the modeling process. Table 1 shows more details regarding the operationalization of system engagement.

**Table 1:** Mobile health (mHealth) engagement across 3 communication levels on the studied intervention app for people with HIV and substance use disorders. Screenshots from the app interface are shown in Figures 3-6.

Engagement variables: communication level and message mode			Operationalization		App interface
Overall			Count of clicks per user per day on specific app page(s)		Home Page
**Network**					Discussion Forum
	Message reception	App page indicates the user is viewing a message/comment posted on the discussion forum			
	Message expression	App page indicates the user is creating, saving, or editing a message/comment posted on the discussion forum			
**Dyadic**					Private Messaging
	Message reception	App page indicates the user is viewing a private one-on-one message thread			
	Message expression	App page indicates the user is creating, saving, or editing a private one-on-one message			
**Intraindividual**					My Motivation
	Message reception	App page indicates the user is viewing a self-written journal entry saved in the past			
	Message expression	App page indicates the user is composing a new journal entry			

**Figure 3:**
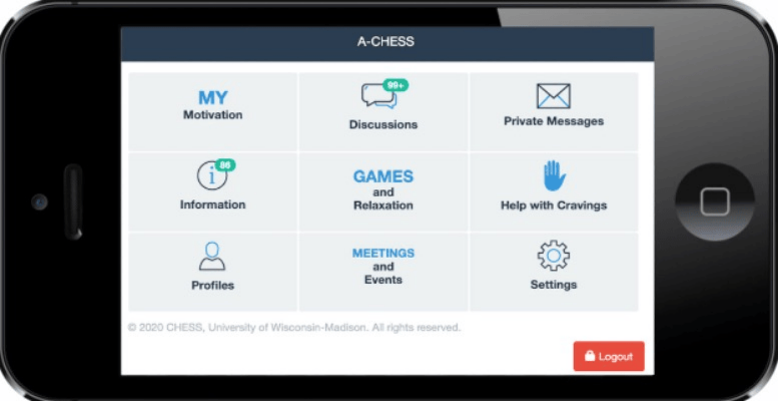
Home page of app interface.

**Figure 4:**
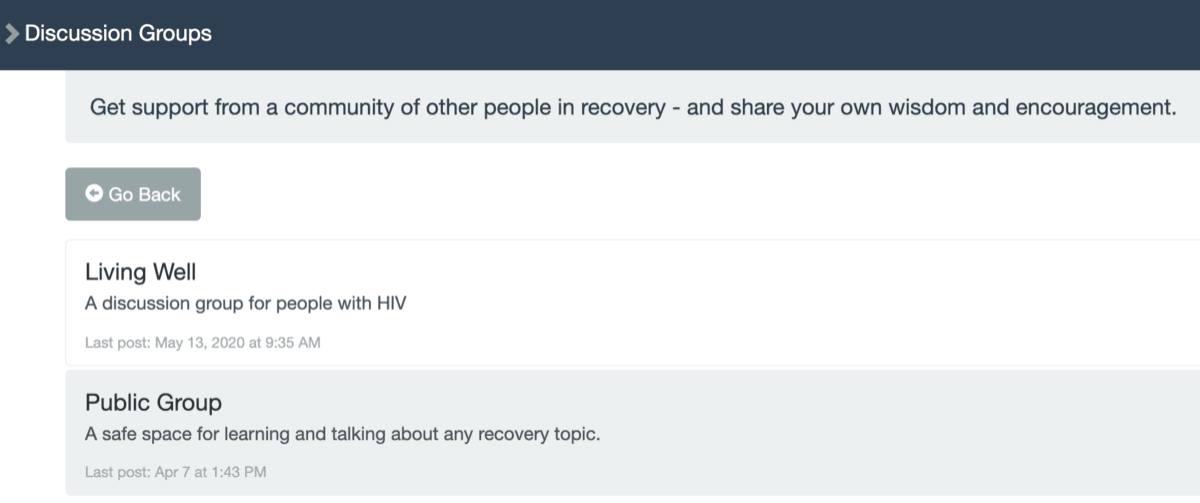
Discussion forum of app interface.

**Figure 5:**
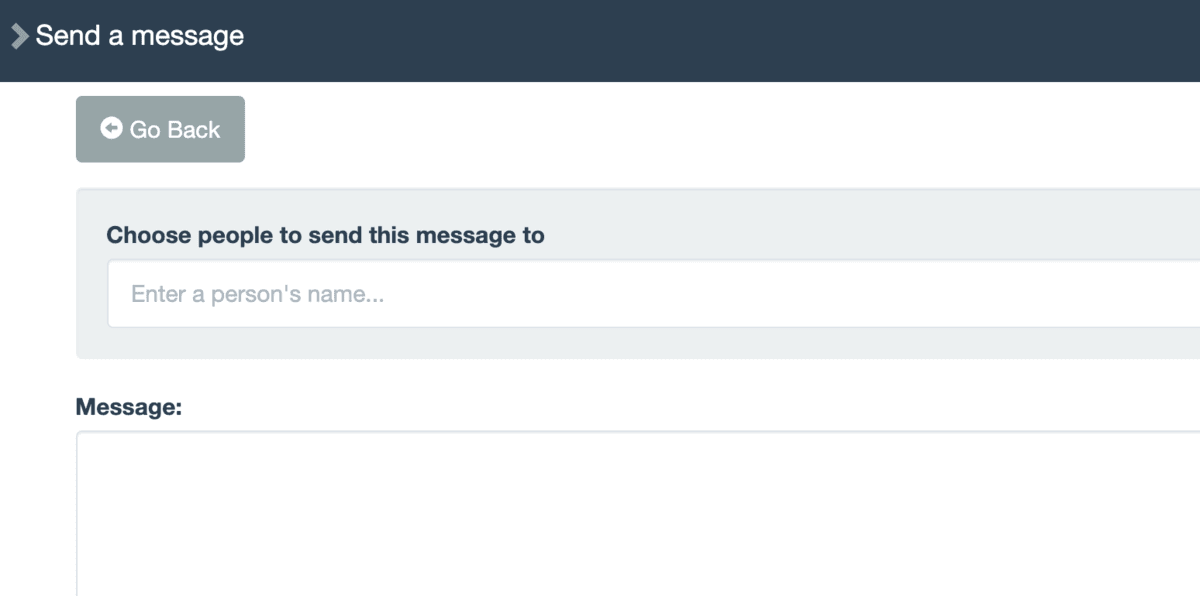
Private messaging on app interface.

**Figure 6:**
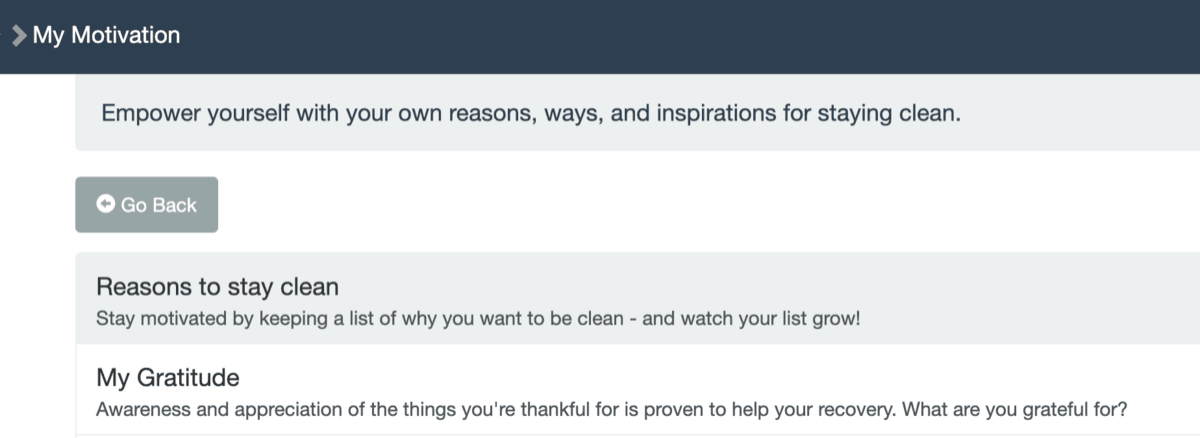
"My Motivation" section of app interface.

#### Medication Adherence

The app administered weekly surveys prompting participants to report the frequency of missed doses of HIV medications within the past week. Responses, ranging from 0-7, indicating the number of missed doses, were then reverse-coded with higher values indicating stronger adherence to the medication regimen.

#### Substance Use

The app administered weekly surveys presenting participants with 4 options (ie, heroin, opioid, alcohol, and stimulants) and asked, “Which of the following, if any, have you used in the last 7 days? (Check all that apply).” To capture whether users engaged with any substance use, checked options were encoded as binary variables. Heroin and opioids were combined as one category. Hence, 3 substance use variables were created to measure the recent use of heroin or opioids, alcohol, and stimulants.

#### Confidence in HIV Management

The app administered weekly surveys prompting participants to report their (1) confidence to keep the next appointment with the HIV care provider, (2) confidence to take all HIV medications during the next week, and (3) confidence in the belief that the HIV viral load “is undetectable right now.” Responses on a 7-point Likert scale to the 3 items were averaged (Cronbach α=0.80).

#### Demographics

Gender, race, age, and years of education were acquired through a baseline survey.

### Analytical Strategies

The unit of analysis was each response to the weekly survey provided by the 208 participants. The click-level data were aggregated into 7-day windows lagged 1 week before the survey input. We then transformed the aggregated clicks into the number of days with nonzero clicks (ie, initiation) and the sum of the square root of daily clicks (ie, intensity). As a result, when testing the hypotheses and RQs, the sample size was raised to 681 since the unit of analysis was not merely user based. Instead, the unit of analysis for the modeling process was the weekly check-in record per user (N=681).

The aggregated, transformed, and lagged system engagement variables were treated as predictors (see the *Measurement* section above for more details). System engagement predictors are deemed significant in the sense that they predict medication adherence through the joint mediators (opioid, alcohol, stimulant, and confidence in HIV). To control the temporal autocorrelation, we fit linear regression with CRSEs for H1-H4 (total) and structural equation modeling with CRSEs for H5-H6, which are typically used for controlling the error correlations at the group level. In our context, SEMs with CRSEs adjusted for the within-person temporal autocorrelation between observations for each app user. To answer RQ1-RQ3, a moderator indicating racial groups (Black or White participants) was added to the SEMs. For robustness check, we also specified an SEM in which system use is treated as a single latent component measured by the 12 system use variables. The results exhibited higher model fit and confirmed the association between app use and medication adherence.

### Ethical Considerations

The study was approved by the Institutional Review Board at the University of Wisconsin-Madison (2016-1190) and funded by National Institutes of Health (grant DP2DA042424). Institutional Review Board–approved recruitment flyers were posted in the UW Health Infectious Disease Clinic, Johns Hopkins Moore Clinic, and the ALIVE study clinic during Phase 1 (2017-2018) and at the UW Health Infectious Disease Clinic during Phase 2 (2018-2020). Verbal consent using a brief script was obtained before administering the screening questionnaire. Eligible individuals were informed about the study details and asked to review the informed consent document, which described the risks and benefits of their participation. During the screening, participants who provided informed consent were given the option to install the study app on their mobile devices.

Participants created a username and password and provided the research team with the necessary information to build a new user account on the server. To protect participants’ privacy, app use data were stored in a secure, encrypted database that was accessible only to the researchers. The information shared by the participants was anonymized before analysis. No monetary compensation was provided to participants. More details are available in the published protocol paper [52].

## Results

A total of 173 participants used at least 1 communication feature (network, dyadic, or intraindividual) during the study period and provided weekly check-in data. The resulting cohort (N=173) had a mean age of 46 (SD 11.2) years and was 77.46% (n=134) male and 64.16% (n=111) Black. Overall, 42.2% (73/173) had at least some college-level education, and 60.12% (104/173) reported illegal or street drugs (including marijuana) or prescription medication abuse in the 30 days before joining this study. On average, participants used this mHealth app for a median of 13 (SD 23; range 1-141) days over the 6-month period. The zero-order correlations for all the numerical variables are presented in Table 2.

**Table 2:** Zero-order correlations for the numerical variables included in the structural equation model that examines how the use of mobile health (mHealth) predicts medication adherence (N=173).

Expression types		1	2	3	4	5	6	7	8	9
**1. Medication adherence**										
	*r*	1	0.24	0.02	0.01	0.02	–0.04	0.01	–0.005	0.09
	*P* value	—^a^	<.001	.69	.76	.58	.27	.73	.90	.02
**2. Confidence in HIV management**										
	*r*	0.24	1	0.07	0.04	0.01	0.01	0.06	0.04	0.2
	*P* value	<.001	—	.06	.28	.75	.73	.13	.25	<.001
**3. Intensity of network reception**										
	*r*	0.02	0.07	1	0.7	0.43	0.3	0.37	0.2	–0.07
	*P* value	.69	.06	—	<.001	<.001	<.001	<.001	<.001	<.001
**4. Intensity of network expression**										
	*r*	0.01	0.04	0.70	1	0.36	0.20	0.25	0.11	–0.09
	*P* value	.76	.28	<.001	—	<.001	<.001	<.001	<.001	<.001
**5. Intensity of intraindividual reception**										
	*r*	0.02	0.01	0.43	0.36	1	0.62	0.41	0.51	0.04
	*P* value	.58	.75	<.001	<.001	—	<.001	<.001	<.001	.08
**6. Intensity of intraindividual expression**										
	*r*	–0.04	0.01	0.30	0.20	0.62	1	0.34	0.41	0.06
	*P* value	.27	.73	<.001	<.001	<.001	—	<.001	<.001	.006
**7. Intensity of dyadic reception**										
	*r*	0.01	0.06	0.37	0.25	0.41	0.34	1	0.54	–0.03
	*P* value	.73	.13	<.001	<.001	<.001	<.001	—	<.001	.21
**8. Intensity of dyadic expression**										
	*r*	–0.005	0.04	0.20	0.11	0.51	0.41	0.54	1	0.07
	*P* value	.90	.25	<.001	<.001	<.001	<.001	<.001	—	<.001
**9. Age**										
	*r*	0.09	0.20	–0.07	–0.09	0.04	0.06	–0.03	0.07	1
	*P* value	.02	<.001	<.001	<.001	.08	.006	.21	<.001	—

^a^Not applicable.

### Total Effects of System Use on Medication Adherence (H1-H4)

The total effects of the initiation and intensity of system use, either by message mode or by communication level (12 variables in total), were not significant in predicting medication adherence, except for the initiation of intraindividual reception (standardized β coefficient = 0.56, 95% CI 0.30-0.82; *P*<.001). Only part of H1 was supported. Nonetheless, the linear regression model suggested that the intensity metrics (eg, network reception, network expression, and dyadic reception) showed a more consistently positive association with medication adherence than initiation metrics. The divergent directions between predictors and the outcome led to the subsequent mediation tests to unpack these seemingly countervailing effects. For this reason, we use SEMs to decompose the mediating effects of the proposed theoretical mechanism [55,56]. Coefficient estimates for all system use variables in H1-H4 are reported in Multimedia Appendix 1.

### Indirect Effects Through Substance Use and Confidence in HIV Management (H5-H6)

The mediation model based on the SEM showed an adequate model fit (^2^_6_=19.70, *P*=.003; comparative fit index=0.824; and standardized root mean squared residual=0.018). Substance use and confidence in HIV management jointly mediated the relationship between 4 types of system use and medication adherence. Through these joint mediators, 3 system use predictors indirectly predicted improved medication adherence: the intensity of dyadic reception (standardized β coefficient=0.036, 95% CI 0.01-0.06; *P*=.01), the intensity of dyadic expression (standardized β coefficient=0.067, 95% CI 0.01-0.13; *P*=.03), and the intensity of network expression (standardized β coefficient=0.039, 95% CI 0.01-0.07; *P*=.02). Notably, the intensity of intraindividual reception predicted worsened medication adherence through the joint mediators (standardized β coefficient=–0.043, 95% CI –0.086 to –0.001; *P*=.05).

**Figure 1:**
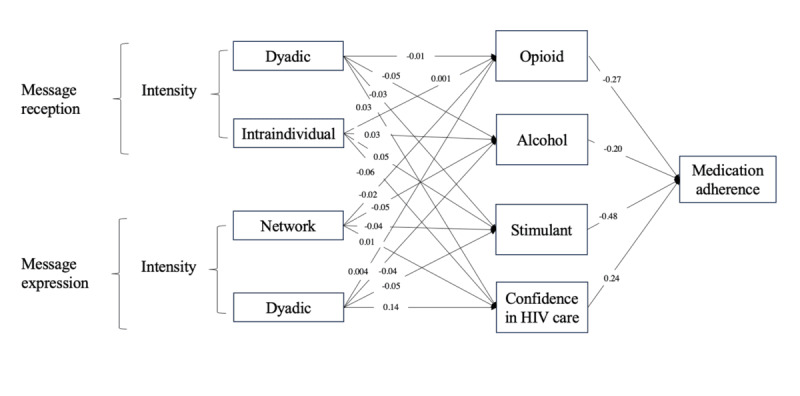
Mediation model showing mobile health (mHealth) engagement predicting medication adherence by substance use and confidence in HIV management (all participants).

**Figure 2:**
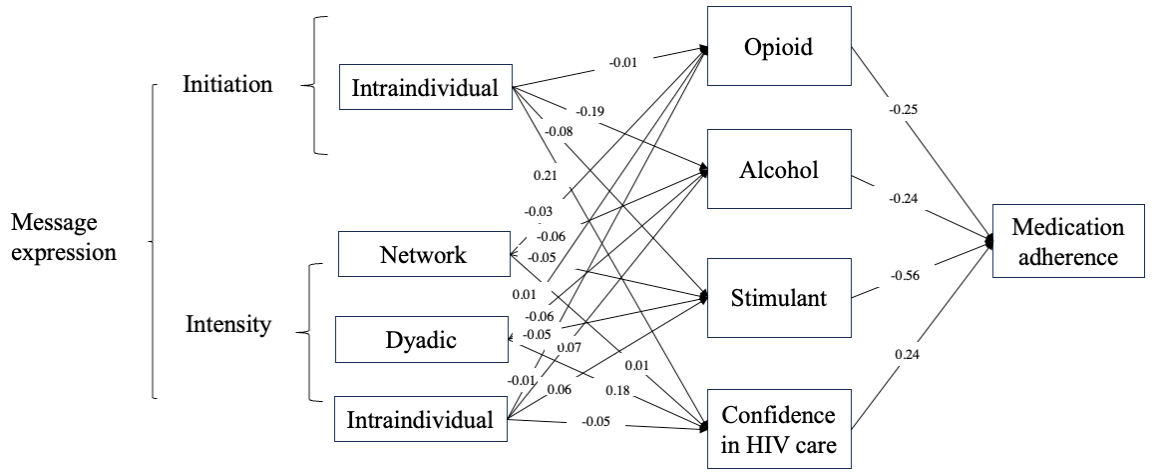
Mediation model showing mobile health (mHealth) engagement predicting medication adherence by substance use and confidence in HIV management (Black participants only).

Figure 1 demonstrates the estimated indirect pathways between significant system engagement predictors and medication adherence through the 4 joint mediators by decomposing the indirect effects into the constituting a-paths (linking engagement metrics to mediators) and b-paths (linking mediators to medication adherence outcomes). For clarity, only statistically significant predictors are presented in the diagram. The 4 listed predictors are significant in that they predict medication adherence via the joint mediators. This presentation rule also applies to Figure 2. All the mediators were allowed to covary with each other. Full estimates of the coefficients of both the joint and individual mediators are reported in Multimedia Appendix 2.

### Racial Differences (RQ1-RQ3)

Race was entered as a binary moderator (ie, whether the users identified themselves as Black) to the SEM. The moderated mediation SEM showed an adequate model fit (*^2^*_6_=17.22, *P*=.009; comparative fit index=0.929; and standardized root mean squared residual=0.011). Subgroup-specific estimates were then extracted from this moderated mediation model.

For Black users (RQ1), results suggest that substance use and confidence in HIV management also jointly mediated the relationship between system engagement and medication adherence (more details are shown in Figure 2 for the diagram and Multimedia Appendix 3 for full estimates). Specifically, through these joint mediators, 3 system use predictors indirectly predicted improved medication adherence: the initiation of intraindividual expression (standardized β coefficient=0.14, 95% CI 0.02=0.27; *P*=.02), the intensity of network expression (standardized β coefficient=0.05, 95% CI 0.01-0.09; *P*=.007), and the intensity of dyadic expression (standardized β coefficient=0.08, 95% CI 0.02-0.15; *P*=.01). Notably, the intensity of intraindividual expression predicted worsened medication adherence through the joint mediators (standardized β coefficient=–0.06, 95% CI –0.11 to –0.01; *P*=.01).

For White participants (RQ2), we lack the evidence that any of the systems use predictors significantly predict medication adherence through joint mediators (more details in Multimedia Appendix 4).

Statistically contrasting Black and White participants, the relationship between system engagement and medication adherence yielded distinct patterns (RQ3). Table 3 reports the difference coefficients for Black versus White participants. Specifically, both the initiation (coefficient=0.22, 95% CI 0.04-0.40; *P*=.02) and intensity (coefficient=–0.07, 95% CI –0.13 to –0.01; *P*=.03) of intraindividual expression differed in their relationship with medication adherence for Black and White participants.

**Table 3:** Difference coefficients estimated for Black and White participants in their mediated relationship between system engagement and medication adherence.

Predictors	Difference coefficient (95% CI), White vs Black	*P* value
Network reception initiation	0.06 (–0.12 to 0.25)	.51
Network reception intensity	–0.02 (–0.05 to 0.0001)	.051
Network expression initiation	0.01 (–0.32 to 0.35)	.93
Network expression intensity	0.03 (–0.07 to 0.13)	.57
Dyadic reception initiation	0.16 (–0.07 to 0.40)	.17
Dyadic reception intensity	–0.03 (–0.08 to 0.02)	.25
Dyadic expression initiation	–0.05 (–0.36 to 0.26)	.75
Dyadic expression intensity	0.08 (–0.06 to 0.22)	.25
Intraindividual reception initiation	0.41 (–0.01 to 0.83)	.06
Intraindividual reception intensity	–0.05 (–0.20 to 0.11)	.56
Intraindividual expression initiation	0.22 (0.04-0.40)	.02
Intraindividual expression intensity	–0.07 (–0.13 to –0.01)	.03

## Discussion

### Principal Findings

Emerging mHealth systems hold promise to enhance medication adherence among people with HIV and SUDs. Despite its potential, previous research has yet to fully explain how mobile technologies relate to medication adherence for this underserved population. Our study examines various aspects of mHealth engagement and their potential impact on medication adherence among people with HIV, overall and by racial groups.

Our findings reveal that most communicative engagement with mHealth does not directly predict medication adherence in people with HIV. Instead, certain metrics of mHealth engagement indirectly predicted medication adherence through substance use and confidence in HIV management as joint mediators. Specifically, at the dyadic level, we observed that greater intensity of message reception and expression (ie, sending and receiving one-on-one messages) were linked to reduced alcohol and stimulant use, as well as increased confidence in HIV management, all of which, in turn, were associated with greater medication adherence, all measured on a weekly basis. That said, the intensity of dyadic expression was associated with greater opioid use, which predicted worsened medication adherence. At the network level, greater intensity of message expression (ie, posting on the discussion forum) was related to decreased opioid, alcohol, and stimulant use, as well as increased confidence in management, all of which predicted improved medication adherence. In contrast, at the intraindividual level, a higher intensity of message reception (ie, reviewing past saved journal entries) was associated with elevated opioid, alcohol, and stimulant use, as well as decreased confidence in management, all of which signaled reduced medication adherence.

### Comparison With Previous Work

Our study contributes to the growing body of mHealth research by disentangling system use along several significant dimensions, including message expression versus reception, communication levels, and initiation versus intensity, as noted in previous literature [14,16]. The findings regarding the overall participants highlight the critical role of message intensity, rather than initiation, in shaping mHealth engagement and its impact on medication adherence. This underscores the importance of considering not only habitual use but also the depth of engagement for people with HIV and SUDs to improve ART medication adherence.

Furthermore, our study underscores the need to account for all 3 communication levels in health interventions, especially when juxtaposing each level with message expression versus reception in both design and analysis. For example, our results highlight that network expression (ie, posting on the discussion forum) was associated with improved health outcomes, while network reception (ie, browsing the discussion forum) may not yield the same benefit. This finding aligns with previous studies, which have indicated that individuals who actively engage in expressing themselves within online communities, often referred to as “expressors,” tend to perceive more benefits [57,58]. In addition, providing emotional support in such interactions can also be beneficial for the message senders [59,60], enhancing their skills in developing coping strategies [61]. Another critical implication of our research is that patients who focus on intraindividual communication may signal potential challenges in SUDs and, eventually, in their medication adherence. This insight serves as a valuable indicator for health care providers to identify individuals who may require additional support and intervention to ensure the success of their medication regimens.

This study also contributes to our understanding of racial differences in their engagement with and outcomes of mHealth technologies. We observed notable distinctions between Black and White participants in how mHealth system engagement may (or may not) be associated with their medication adherence despite the fact that they were using the same mHealth app. Among Black participants, our results highlight the importance of message expression (Figure 2) as message expression at different communication levels, constituting a total of 12 distinct pathways, emerged as a set of critical predictors. These expression predictors, rather than any reception predictors, connect with both risk and protective factors, which, in turn, play a pivotal role in predicting medication adherence. In addition, the 2 racial groups also differ in the role played by intraindividual expression on medication adherence (Table 3). These disparities underscore the need for a nuanced, race-sensitive approach to medical interventions and health care support for people with HIV. Tailoring strategies based on these findings may help address racial disparities in health outcomes.

The third contribution of this study is methodological, where we introduced 2 engagement metrics based on longitudinal and timestamped system log data: daily initiation and intensity of system use within each active session. We tested the predictive power of these metrics using linear regression and SEMs with CRSEs to control for within-person autocorrelation and group-level error correlations. This approach can guide health practitioners in tailoring interventions for those showing less intensive use, revealing which app features supported ART medication adherence. Another avenue to explore is tracking the evolution of these 2 engagement types over time. By examining the fluctuations in mHealth engagement, we can extract crucial insights about the dynamic characteristics of these interventions. Attention to these dynamics may help further tailor the design of mHealth interventions to serve the evolving needs of people with HIV living with SUD. These insights might also inform the roles of health care professionals in promoting medication adherence among this population.

### Practical Implications

As the adoption of mHealth interventions for improving medication adherence among people with HIV and SUDs increases, the findings from this study may provide insights into how to enhance their impact. Clinicians should encourage patients to participate in one-on-one messaging (dyadic communication) and actively post on discussion forums (network expression). In addition, monitoring system use patterns such as frequent reviewing of past journal entries and sending one-on-one messages may serve to identify patients in need of additional support. The findings from this study also indicate it is important to tailor mHealth interventions for specific subgroups. For instance, Black participants who showed distinct usage patterns may be more influenced by their expression across different communication levels.

Finally, our findings provide useful guidance for app developers. App developers should prioritize features that promote expressive behaviors in one-on-one messaging and discussion forums, such as reminders and notifications. Furthermore, developers should implement a monitoring and alert system to detect frequent reviewing of past journal entries, enabling timely connection of patients with health care providers for necessary support.

### Limitations

We acknowledge this study has several limitations. First, this research did not consider the content of messages at the network, dyadic, or intraindividual level, ignoring the nature of the communication that may play a buffering role. Future research should examine message content to provide a deeper understanding of why, for instance, message reception at the intraindividual level negatively predicted medication adherence through the tested mediators. Second, some participants did not consistently complete the weekly check-in surveys, leading to missing data.

However, given that our unit of analysis is the user weekly survey, our analysis does not require that the surveys be completed at regular intervals. We model each user survey and allow for arbitrary correlation between surveys by the same user through robust SEs clustered on the user. Due to this approach, when users did not take a survey on a given week and failed to provide the dependent variable and all mediators, this missed survey is not considered missing data in the analysis in a practical sense. Future research may investigate the possibilities of data imputation techniques for simulation purposes [62]. It is also important to note that our sample is relatively small and does not represent the diverse population of people with HIV living with SUDs. Those who were willing to participate in our study were likely to have greater motivations to maintain medication adherence, potentially biasing results. Future research should aim to include a more diverse and larger sample to enhance generalizability.

### Conclusions

To summarize, this study articulates the different aspects of mHealth engagement and emphasizes the importance of the intensity of engagement over initiation for promoting medication adherence in people with HIV and SUDs. The findings highlight the need for nuanced patient engagement strategies and targeted interventions due to the unique usage patterns and associations with ART medication adherence of Black and White participants.

## Appendix

Multimedia Appendix 1 Effect estimates of system use on medication adherence (H1-H4).

Multimedia Appendix 2 Joint and separate indirect effects of all mediators connecting system engagement predictors and medication adherence (all participants).

Multimedia Appendix 3 Joint and separate indirect effects of all mediators connecting system engagement predictors and medication adherence (Black participants).

Multimedia Appendix 4 Joint and separate indirect effects of all mediators connecting system engagement predictors and medication adherence (White participants).

## Abbreviations

ART: antiretroviral therapy
CRSE: cluster-robust SE
EAM: electronic adherence monitoring
mHealth: mobile health
RQ: research question
SEM: structure equation model
SUD: substance use disorders

## Data Availability

The datasets generated during this study are not publicly available due to the sensitive nature of the peer-to-peer communication data in recovery support forums and the fact that the participants did not consent to the broad sharing of the research data. However, numerical data underlying the regression analyses are available from the corresponding author on reasonable request.
