# Supplementary material for: mHealth Engagement for Antiretroviral Medication Adherence Among People With HIV and Substance Use Disorders: Observational Study
Source: J Med Internet Res. 2024 Dec 20;26:e57774. doi: 10.2196/57774 (PMC11699505; doi:10.2196/57774)
Supplement: Multimedia Appendix 1 [file jmir_v26i1e57774_app1.docx]

**Multimedia Appendix 1. Effect Estimates of System Use on Medication Adherence (H1-H4)**

|  | *Medication Adherence* | | |
| --- | --- | --- | --- |
| *Predictors* | *β* | 95% CI | *P value* |
| Network reception initiation | -0.14 | (-0.42, 0.14) | .32 |
| Network reception intensity | 0.02 | (-0.003, 0.04) | .10 |
| Network expression initiation | -0.25 | (-0.74, 0.24) | .31 |
| Network expression intensity | 0.05 | (-0.02, 0.11) | .16 |
| Dyadic reception initiation | -0.20 | (-0.51, 0.11) | .20 |
| Dyadic reception intensity | 0.07 | (-0.003, 0.14) | .06 |
| Dyadic expression initiation | -0.29 | (-0.77, 0.19) | .25 |
| Dyadic expression intensity | 0.07 | (-0.09, 0.23) | .41 |
| Intraindividual reception initiation | 0.56 | (0.30, 0.82) | <.001 |
| Intraindividual reception intensity | -0.08 | (-0.19, 0.04) | .20 |
| Intraindividual expression initiation | 0.07 | (-0.14, 0.27) | .52 |
| Intraindividual expression intensity | -0.05 | (-0.13, 0.04) | .26 |
| *Note*. The 95% confidence interval is in the parentheses. | | | |
