# Supplementary material for: mHealth Engagement for Antiretroviral Medication Adherence Among People With HIV and Substance Use Disorders: Observational Study
Source: J Med Internet Res. 2024 Dec 20;26:e57774. doi: 10.2196/57774 (PMC11699505; doi:10.2196/57774)
Supplement: Multimedia Appendix 2 [file jmir_v26i1e57774_app2.docx]

**Multimedia Appendix 2. Joint and separate indirect effects of all mediators connecting system engagement predictors and medication adherence (all participants)**

|  | *Joint Mediators* | | *Opioids* | | *Alcohol* | | *Stimulants* | | *Confidence in HIV Management* | |
| --- | --- | --- | --- | --- | --- | --- | --- | --- | --- | --- |
| *Predictors* | *β*  *(95% CI)* | *P value* | *β*  *(95% CI)* | *P value* | *β*  *(95% CI)* | *P value* | *β*  *(95% CI)* | *P value* | *β*  *(95% CI)* | *P value* |
| Network reception initiation | 0.02  (-0.05, 0.09 ) | .55 | 0.01  (-0.01, 0.02) | .44 | 0.02  (-0.02, 0.05) | .40 | 0.02  (-0.02, 0.05) | .40 | -0.01  (-0.06, 0.03) | .55 |
| Network reception intensity | -0.0002  (-0.01, 0.012 ) | .98 | -0.001  (-0.004 , 0.001) | .31 | -0.0004  (-0.01, 0.004) | .88 | -0.003  (-0.01, 0.003) | .29 | 0.01  (-0.003, 0.01) | .21 |
| Network expression initiation | -0.11  (-0.25, 0.024) | .11 | -0.02  (-0.06, 0.02) | .26 | -0.04  (-0.10, 0.02) | .18 | -0.01  (-0.08, 0.06) | .81 | -0.04  (-0.13, 0.06) | .45 |
| Network expression intensity | 0.04*  (-0.01, 0.07) | .020 | 0.01  (-0.01, 0.02) | .24 | 0.01  (-0.01, 0.03) | .27 | 0.02  (-0.0004, 0.04) | .06 | 0.004  (-0.01, 0.02) | .54 |
| Dyadic reception initiation | -0.06  (-0.15, 0.03) | .16 | -0.01  (-0.02, 0.01) | .47 | -0.03  (-0.07, 0.02) | .22 | -0.05  (-0.12, 0.02) | .14 | 0.02  (-0.02, 0.06) | .23 |
| Dyadic reception intensity | 0.04*  (0.01, 0.06) | .014 | 0.001  (-0.003, 0.01) | .55 | 0.01  (-0.004, 0.03) | .16 | 0.02  (-0.004, 0.04) | .13 | 0.008  (-0.01, 0.02) | .21 |
| Dyadic expression initiation | -0.11  (-0.26, 0.05) | .18 | 0.01  (-0.01, 0.04) | .27 | -0.01  (-0.06, 0.04) | .72 | -0.03  (-0.14, 0.072) | .55 | -0.08  (-0.21, 0.05) | .23 |
| Dyadic expression intensity | 0.07*  (0.01, 0.13) | .026 | -0.001  (-0.01, 0.004) | .67 | 0.01  (-0.01, 0.03) | .36 | 0.03  (-0.01, 0.06) | .19 | 0.03  (-0.01, 0.08) | .14 |
| Intraindividual reception initiation | 0.04  (-0.08, 0.16) | .53 | 0.001  (-0.01, 0.01) | .82 | 0.01  (-0.03, 0.05) | .57 | 0.02  (-0.03, 0.07) | 0.33 | 0.003  (-0.09, 0.10) | .95 |
| Intraindividual reception intensity | -0.04*  (-0.09, 0.001) | .047 | -0.0001  (-0.01, 0.01) | .96 | -0.01  (-0.02, 0.01) | .33 | -0.02  (-0.05, 0.001) | .06 | -0.01  (-0.04, 0.01) | .22 |
| Intraindividual expression initiation | 0.40  (-0.04, 0.12) | .35 | 0.001  (-0.004, 0.01) | .76 | 0.02  (-0.01, 0.05) | .24 | 0.01  (-0.03, 0.05) | .52 | 0.01  (-0.05, 0.06) | .77 |
| Intraindividual expression intensity | -0.03  (-0.07, 0.01) | .14 | 0.0003  (-0.003, 0.003) | .86 | -0.01  (-0.02, 0.01) | .28 | -0.02  (-0.04, 0.01) | .15 | -0.003  (-0.02, 0.01) | .60 |
| *Note*. The 95% confidence interval is in the parentheses. **p* <. 05 ***p* <. 01 ****p* <. 01 | | | | | | | | | | |
