# Supplementary material for: mHealth Engagement for Antiretroviral Medication Adherence Among People With HIV and Substance Use Disorders: Observational Study
Source: J Med Internet Res. 2024 Dec 20;26:e57774. doi: 10.2196/57774 (PMC11699505; doi:10.2196/57774)
Supplement: Multimedia Appendix 3 [file jmir_v26i1e57774_app3.docx]

**Multimedia Appendix 3. Joint and separate indirect effects of all mediators connecting system engagement predictors and medication adherence (Black participants).**

|  | *Joint Mediators* | | *Opioids* | | *Alcohol* | | *Stimulants* | | *Confidence in HIV Management* | |
| --- | --- | --- | --- | --- | --- | --- | --- | --- | --- | --- |
| *Predictors* | *β*  *(95% CI)* | *P value* | *β*  *(95% CI)* | *P value* | *β*  *(95% CI)* | *P value* | *β*  *(95% CI)* | *P value* | *β*  *(95% CI)* | *P value* |
| Network reception initiation | 0.07  (-0.03, 0.18) | .15 | 0.003  (-0.01, 0.02) | .73 | 0.04  (-0.01, 0.09) | .12 | 0.05*  (0.01, 0.11) | .026 | -0.02  (-0.08, 0.04) | .48 |
| Network reception intensity | -0.01  (-0.02, 0.005) | .20 | -0.001  (-0.004, 0.002) | .41 | -0.005  (-0.01, 0.001) | .12 | -0.007*  (-0.01, -0.0006) | .032 | 0.004  (-0.01, 0.01) | .38 |
| Network expression initiation | -0.11  (-0.30, 0.10) | .32 | -0.04  (-0.89, 0.19) | .20 | 0.003  (-0.04, 0.05) | .90 | -0.04  (-0.14, 0.06) | .46 | -0.03  (-0.17, 0.10) | .62 |
| Network expression intensity | 0.05*  (0.01, 0.09) | .01 | 0.04  (-0.02, 0.11) | .19 | 0.02  (-0.006, 0.04) | .16 | 0.02*  (0.002, 0.05) | .035 | 0.003  (-0.01, 0.01) | .56 |
| Dyadic reception initiation | 0.0002  (-0.10, 0.11) | 1.00 | -0.01  (-0.41, 0.24) | .61 | -0.01  (-0.042, 0.03) | .80 | -0.03  (-0.10, 0.04) | .44 | 0.05  (-0.02, 0.11) | .20 |
| Dyadic reception intensity | 0.02  (-0.01, 0.05) | .28 | 0.002  (-0.006, 0.009) | .66 | 0.005  (-0.006, 0.02) | .38 | 0.01  (-0.01, 0.03) | .45 | 0.001  (-0.02, 0.02) | .89 |
| Dyadic expression initiation | -0.09  (-0.26, 0.07) | .27 | 0.02  (-0.02, 0.06) | .34 | -0.01  (-0.07, 0.05) | .72 | -0.01  (-0.09, 0.06) | .72 | -0.09  (-0.22, 0.05) | .20 |
| Dyadic expression intensity | 0.08*  (0.02, 0.15) | .01 | -0.003  (-0.01, 0.01) | 0.54 | 0.01  (-0.008, 0.04) | .20 | 0.02  (-0.01, 0.06) | .16 | 0.04  (-0.005, 0.09) | .08 |
| Intraindividual reception initiation | 0.11  (-0.01, 0.23) | .07 | 0.002  (-0.01, 0.02) | .82 | 0.03  (-0.02, 0.08) | .22 | 0.03  (-0.02, 0.08) | .24 | 0.05  (-0.02, 0.12) | .18 |
| Intraindividual reception intensity | -0.02  (-0.07, 0.03) | .41 | -0.001  (-0.01, 0.01) | .77 | 0.002  (-0.01, 0.01) | .68 | -0.01  (-0.04, 0.02) | .43 | -0.01  (-0.04, 0.02) | .40 |
| Intraindividual expression initiation | 0.14*  (0.02, 0.27) | .02 | 0.003  (-0.003, 0.01) | .28 | 0.05  (-0.01, 0.10) | .08 | 0.04  (-0.01, 0.10) | .12 | 0.05  (-0.02, 0.12) | .18 |
| Intraindividual expression intensity | -0.06*  (-0.11, -0.01) | .01 | 0.001  (-0.003, 0.005) | .49 | -0.02  (-0.04, 0.003) | .10 | -0.03*  (-0.07, -0.002) | .036 | -0.01  (-0.03, 0.003) | .12 |
| *Note*. The 95% confidence interval is in the parentheses. **p* <. 05 ***p* <. 01 ****p* <. 01 | | | | | | | | | | |
