# Supplementary material for: mHealth Engagement for Antiretroviral Medication Adherence Among People With HIV and Substance Use Disorders: Observational Study
Source: J Med Internet Res. 2024 Dec 20;26:e57774. doi: 10.2196/57774 (PMC11699505; doi:10.2196/57774)
Supplement: Multimedia Appendix 4 [file jmir_v26i1e57774_app4.docx]

**Multimedia Appendix 4. Joint and separate indirect effects of all mediators connecting system engagement predictors and medication adherence (White participants).**

|  | *Joint Mediators* | | *Opioids* | | *Alcohol* | | *Stimulants* | | *Confidence in HIV Management* | |
| --- | --- | --- | --- | --- | --- | --- | --- | --- | --- | --- |
| *Predictors* | *β*  *(95% CI)* | *P value* | *β*  *(95% CI)* | *P value* | *β*  *(95% CI)* | *P value* | *β*  *(95% CI)* | *P value* | *β*  *(95% CI)* | *P value* |
| Network reception initiation | 0.02  (-0.12, 0.15) | .82 | 0.001  (-0.003, 0.01) | .55 | 0.009  (-0.04, 0.06) | .72 | -0.04  (-0.12, 0.05) | .37 | 0.04  (-0.02, 0.10) | .18 |
| Network reception intensity | 0.01  (-0.004, 0.03) | .12 | 0.001  (-0.002, 0.004) | .56 | 0.005  (-0.002, 0.01) | .18 | 0.006  (-0.009, 0.02) | .46 | 0.003  (-0.007, 0.01) | .53 |
| Network expression initiation | -0.12  (-0.40, 0.16) | .41 | -0.003  (-0.01, 0.005) | .46 | -0.12  (-0.26, 0.03) | .11 | 0.08  (-0.08, 0.23) | .33 | -0.07  (-0.23, 0.08) | .36 |
| Network expression intensity | 0.03  (-0.07, 0.12) | .59 | -0.0002  (-0.003, 0.003) | .92 | 0.009  (-0.03, 0.05) | .64 | 0.004  (-0.03, 0.04) | .80 | 0.01  (-0.03, 0.05) | .53 |
| Dyadic reception initiation | -0.16  (-0.35, 0.03) | .10 | 0.002  (-0.004, 0.008) | .47 | -0.06  (-0.15, 0.02) | .14 | -0.09  (-0.23, 0.05) | .20 | -0.009  (-0.11, 0.09) | .85 |
| Dyadic reception intensity | 0.05  (-0.002, 0.10) | .061 | 0.0009  (-0.003, 0.004) | .62 | 0.02  (-0.006, 0.04) | .16 | .02  (-0.02, 0.06) | .30 | 0.01  (-0.02, 0.04) | .45 |
| Dyadic expression initiation | -0.04  (-0.31, 0.22) | .75 | .005  (-0.009, 0.02) | .50 | -0.11  (-0.31, 0.09) | .28 | 0.02  (-0.31, 0.36) | .89 | 0.04  (-0.24, 0.32) | .78 |
| Dyadic expression intensity | -0.002  (-0.13, 0.12) | .98 | -0.004  (-0.02, 0.008) | .51 | .05  (-0.07. 0.17) | 39 | -0.02  (-0.12, 0.08) | .73 | -0.03  (-0.17, 0.11) | .66 |
| Intraindividual reception initiation | -0.30  (-0.70, 0.07) | .12 | 0.008  (-0.01, 0.03) | .49 | -0.12  (-0.26, 0.02) | .088 | -0.02  (-0.17, 0.12) | .75 | -0.16  (-0.39, 0.07) | .18 |
| Intraindividual reception intensity | 0.03  (-0.13, 0.18) | .72 | -0.004  (-0.02, 0.008) | .51 | 0.04  (-0.02, 0.10) | .20 | -0.02  (-0.14, 0.09) | .67 | 0.02  (-0.06, 0.09) | .69 |
| Intraindividual expression initiation | -0.08  (-0.19, 0.04) | .18 | .002  (-.01, 0.01) | .80 | -0.009  (-0.05, 0.03) | .68 | -0.02  (-0.08, 0.04) | .58 | -0.05  (-0.13, 0.03) | .19 |
| Intraindividual expression intensity | 0.004  (-0.03, 0.04) | .80 | -0.004  (-0.02, 0.009) | .54 | 0.005  (-0.008, 0.02) | .48 | -0.003  (-0.02, 0.02) | .79 | .007  (-0.008, -.02) | .38 |
| *Note*. The 95% confidence interval is in the parentheses. **p* <. 05 ***p* <. 01 ****p* <. 01 | | | | | | | | | | |
